# Bowel Obstruction After Gastric Bypass Surgery: A Narrative Review

**DOI:** 10.7759/cureus.75386

**Published:** 2024-12-09

**Authors:** Athul Pratheesh George, Khaled W Erekat, Cara Mohammed, Navrooh Kaur, Arshpreet Kaur, Shushrusha Adhikari, Aaliya Rahman, Himayath Lakshmannagari, Cesar D Tavera-Castaño, Mounika Vallakati, Sehajpreet Kaur, Zahra Nazir

**Affiliations:** 1 Nephrology, Amala Institute of Medical Sciences, Thrissur, IND; 2 General Surgery, King Hussein Medical Center, Amman, JOR; 3 Orthopedic Surgery, Sangre Grande Hospital, Sangre Grande, TTO; 4 Medicine, Avalon University School of Medicine, Willemstad, CUW; 5 Surgery, Private Practice, Punjab, IND; 6 Internal Medicine, Kathmandu Medical College, Kathmandu, NPL; 7 Internal Medicine, Dr. D. Y. Patil Medical College, Hospital & Research Centre, Pune, IND; 8 General Surgery, Narayana Medical College, Tirupati, IND; 9 Surgery, Universidad Nacional de Colombia, Bogotá, COL; 10 Internal Medicine, Prathima Institute of Medical Sciences, Karimnagar, IND; 11 Medicine/Surgery/Emergency Medicine/Obstetrics and Gynecology, Punjab Institute of Medical Sciences, Jalandhar, IND; 12 Internal Medicine, Combined Military Hospital, Quetta, PAK

**Keywords:** bariatric surgery, comorbid obesity, hernia, intra-abdominal adhesions, small bowel obstruction

## Abstract

Bowel obstruction is a common complication that can affect patients due to different factors, including after a history of gastric bypass surgery. This review was conducted by searching the literature using both PubMed and Google Scholar for articles relating to bowel obstructions. Fifty-six articles were found after applying inclusion and exclusion criteria. Based on the literature search, there are many causes of bowel obstruction, the most common of which include internal hernias, adhesions, and intussusception. Regarding management strategies, surgery is usually the definitive treatment method, but other studies favor a non-surgical approach depending on the specific case.

Bowel obstruction is a significant complication that can occur post gastric bypass surgery. This review sheds light on various causes of bowel obstruction, including the possible treatment options, prevention of bowel obstruction that could be achieved by implementing different surgical techniques, the importance of postoperative monitoring, and timely intervention.

## Introduction and background

Obesity has been a problem that plagued us for centuries, and, unfortunately, it is only getting worse. The World Health Organization (WHO) proclaimed obesity a global epidemic in 1997, and the American Medical Association followed suit by declaring obesity a disease that requires treatment in 2013 [1,2]. To combat this disease, surgical methods have been proposed by Dr. Mason in the 1960s after noticing significant weight loss in patients undergoing gastrectomy for ulcers, marking the first bariatric surgery [3,4].

Bariatric surgery, a weight reduction procedure also known as metabolic surgery, is divided into three categories: malabsorptive (deviates from part of the small intestine), restrictive (narrows the stomach), and a combination of both [5]. The bariatric surgeries currently approved to be done in the United States are sleeve gastrectomy, Roux-en-Y gastric bypass (RYGB), adjustable gastric band (AGB), biliopancreatic diversion with duodenal switch (BPD/DS), and single anastomosis duodenal-ileal bypass with sleeve gastrectomy (SADI-S) [5]. This article will shed light on types of surgeries, focusing on RYGB and its complications.

An estimated 579,000 bariatric surgeries were performed in 2014, making it one of the surgical procedures with the fastest rate of growth across the globe [1]. When skilled surgeons at reputable bariatric clinics carry out the surgery, the risk of serious intraoperative, early postoperative complications and mortality is comparatively low. The total risk of intraoperative complications from bariatric surgery in the modern series is 3.0% for laparoscopic AGB, 5.5% for laparoscopic RYGB, and 7.3% for open RYGB [2]. The rate of intraoperative complications with one-anastomosis gastric bypass (OAGB) has been estimated to be as high as 4.63% in a single-center research and as low as 0.5% in a multicenter study [4].

The operating process, surgical approach (open versus laparoscopic), and other factors affect the risk of a significant complication within 30 days of surgery [5]. Laparoscopic sleeve gastrectomy often has a lower 30-day morbidity rate (0.8% to 5.6% versus 1.4% to 9.4%) than laparoscopic RYGB, and compared to open RYGB, the 30-day morbidity rate with laparoscopic RYGB is lower (3.4% against 7.4%) [6].

As with any other type of surgery, bariatric surgery has certain complications we should look for. Complications after bariatric surgery are divided into early or late complications depending on how much time has passed after the surgery before the complication occurred. Some early complications include gastric perforation or acute dilatation that can be seen after AGB and digestive fistula or hemorrhage after RYGB. Late complications include esophageal dilatation after AGB and internal hernia after RYGB, which is the most common cause of obstruction after RYGB [6]. According to the National Institutes of Health (NIH), a mechanical or functional obstruction that can occur in either the small or large intestine is a bowel obstruction [7]. Currently, small bowel obstruction (SBO) occurs at a rate of 0.2-4.5% after bypass surgery. Compared to 15% of hospital admissions for patients suffering from acute abdominal pain due to bowel obstruction, we can understand how important it is to diagnose and treat the condition adequately [8,9]. This paper will elaborate on the clinical symptoms of bowel obstruction after gastric bypass, the diagnostic modalities and treatment options, and how we can prevent it. The economic impact of bowel obstruction is a significant concern. Recent estimates suggest that direct medical costs related to intestinal obstruction exceed $3 billion, which puts a considerable strain on healthcare setups across the globe [10]. Advanced imaging techniques, such as computed tomography (CT) scans and exploratory laparoscopies, further escalate costs. In cases where emergency surgery is needed, expenses further increase. Apart from direct costs, indirect costs related to bowel obstruction stem from reduced quality of life, lost productivity, and stress related to repeated admissions [11]. This paper will elaborate on the clinical symptoms of bowel obstruction after gastric bypass, the diagnostic modalities and treatment options, and how we can prevent it.

## Review

**Figure 1:**
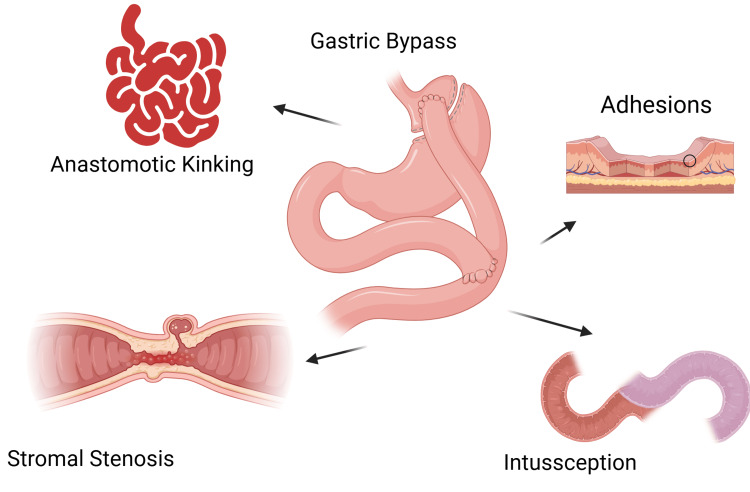
Complications arising secondary to gastric bypass. Figure created using biorender.com.

With a lifetime incidence of 6% to 9.6%, SBO can happen at any point following RYGB [10,11]. Bowel obstruction following laparoscopic surgery is more common (1.8-7.3%) than it is following open gastric bypass surgery (1.3-4%) [12,13]. Misconstruction during RYGB, internal herniation, or adhesion development (in both open and laparoscopic operations) can all cause SBO. Technical errors, such as failing to reapproximate the fascia from a port site or possible internal hernia sites, typically cause early SBO. One of the leading causes of late SBO is internal hernias, which is about 54%, or adhesions, which amounts to around 14% [14]. Adhesions, internal hernias, and intussusceptions can all result in SBO following gastric bypass surgery, and possible complications are depicted in Figure 1 [15].

Internal hernia

**Figure 2:**
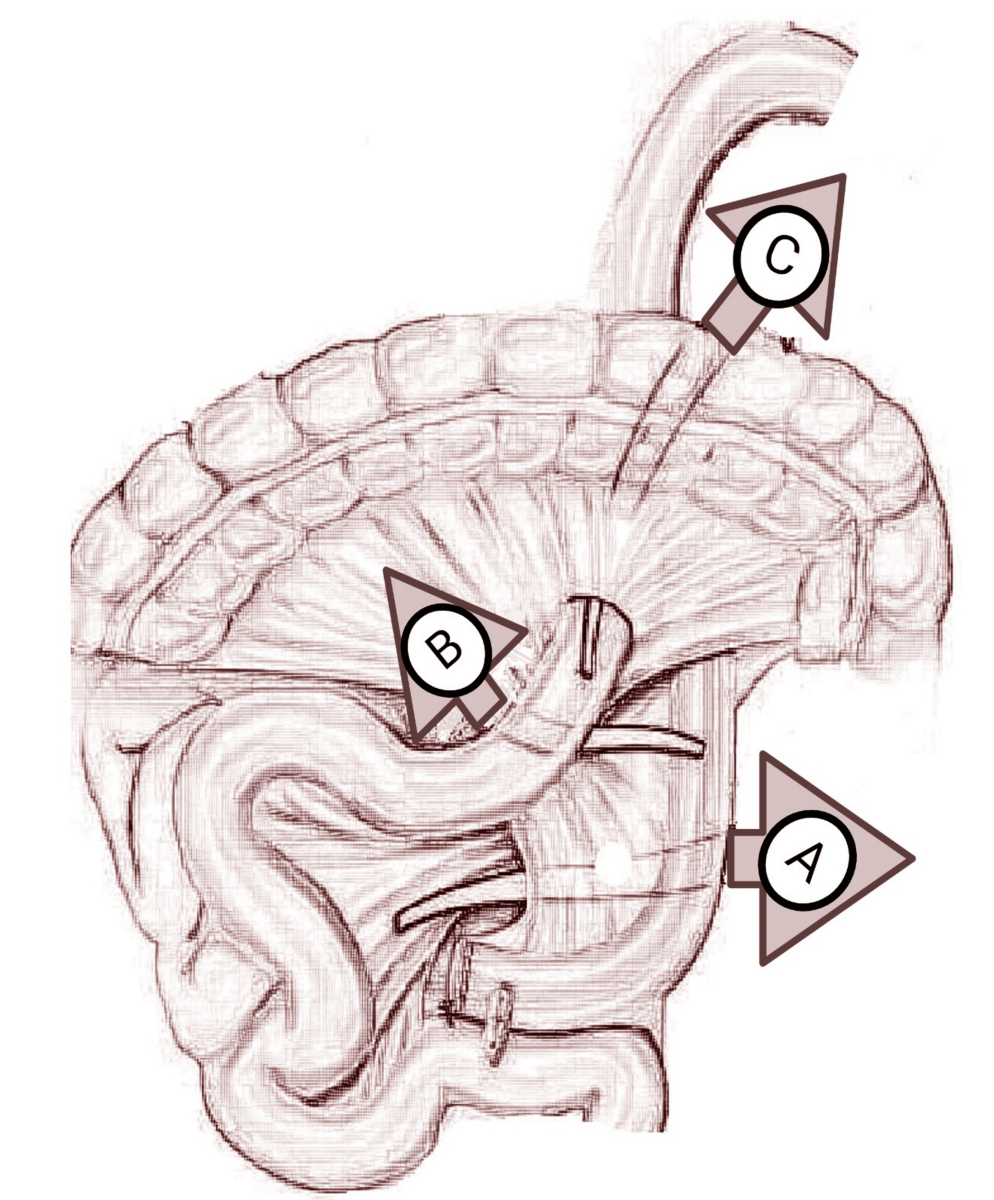
A. Mesenteric defect at the jejunojejunostomy. B. Space between the transverse mesocolon and Roux limb mesentery (i.e., Petersen defect). C. Defect in the transverse mesocolon in patients with a retrocolic Roux limb. Figure created using biorender.com.

Possible gaps that develop during an RYGB can result in SBO, as depicted in Figure 2.

Hernias via the jejunal mesentery can occur below the mesentery of the Roux limb, as well as through the defect below the jejunojejunostomy. An internal hernia following an RYGB may develop posterior to the gastrojejunal anastomosis and across the mesentery of the jejunojejunal anastomosis. An internal hernia may develop through the transverse mesocolon defect and Petersen's defect, which is the gap created by the transverse mesocolon and the Roux limb as they pass through the mesocolon if a retrocolic approach is taken [16]. After RYGB without normal closure of the mesenteric malformations, internal hernias have been seen in 12% of patients [17]. Nonabsorbable sutures should be used to seal any mesenteric abnormalities to lower the risk of internal hernias [18]. After laparoscopic gastric bypass, the transverse mesocolon defect was the site of most internal hernias (44 of 66 in one research) [19]. Although rare, there have been reports of Richter's hernia at the trocar site following laparoscopic RYGB [20,21].

Adhesions

Diffuse adhesions are associated with prolonged hospital stays while band adhesions wrap the intestine in more acute form. Open gastric procedures may cause the creation of upper abdominal adhesions, which secure the bowel and prevent internal hernias via the mesenteric apertures established during the operation [15]. Mesenteric closure raised early postoperative morbidity from jejunojejunostomy kinking (4.3% against 2.8%) but dramatically reduced the risk of reoperation for SBO (6% versus 10% at three years) as compared to non-closure. In research, regular closure of all such abnormalities decreased the risk of small bowel blockage from 6% to 3% [22]. Adhesions were the primary cause of SBO, according to a study by Hwang et al., even though laparoscopic surgery has fewer adhesion forms than open surgery. This could be related to the various adhesion patterns developed during laparoscopic surgery. Adhesions developed following open surgery are typically diffuse and broad-based, while those seen following laparoscopic surgery are typically discrete bands. Single-band adhesions may increase the risk of obstruction by kinking the intestine or acting as a focal point for volvulization [23].

Intussusception

Jejunojejunal intussusception is a rare cause of bowel blockage following gastric bypass surgery [24,25]. Women who have lost a substantial amount of weight are more likely to experience this condition, which is caused by a combination of factors, including a lead point (suture lines, adhesions, and lymphoid hyperplasia), motility difficulties, and abnormal intestinal pacemakers [26-28]. The majority of post-RYGB intussusceptions occur at the jejunojejunostomy site and are retrograde (anti-peristaltic). Intussusception may occur when a jejunojejunostomy is longer than 60 mm [29].

Other causes

SBO at a jejunojejunostomy can occur due to kinking or narrowing of the anastomosis. Kinks typically occur near the transverse staple line, where the enterotomy was closed. Narrowing of the jejunojejunostomy occurs when too much intestinal tissue is stapled during the enterotomy closure [23]. Gastrojejunostomy obstruction is also an infrequent complication of gastric bypass surgery. Stomal stenosis caused by fibrous scarring of the circumferential stoma can sometimes cause the problem. This commonly happens in the first three months after surgery [30].

Clinical presentation and differentiation from other postoperative complications

Diagnosing bowel obstruction requires a strong suspicion. Symptoms of internal herniation are typically ambiguous. Common complaints include nausea, emesis, and stomach pain, typically in the left upper quadrant [15]. Usually unrelated to eating, abdominal pain might be severe, cramping, imprecise, or intermittent. Patients rarely vomit because the stomach pouch is so small. In a study by Husain et al., abdominal discomfort (82.0%) was the most often reported presenting symptom, followed by nausea (48.6%) and vomiting (46.8%). In their study, 13 (27.9%) patients had all three symptoms upon presentation [14]. In cases of stomal stenosis, the patient experiences post-prandial retrosternal pain and regurgitation, progressing to repeated regurgitation of ingested fluids and frothy mucus, and gradually becomes unable to accept oral intake, even liquids. Upper endoscopy is the most effective way to confirm a diagnosis; however, a history is generally sufficient [30-32]. Bilious vomiting occurs when the jejunum becomes obstructed immediately distal to the Roux-Y jejunojejunostomy. Bile in the nasogastric tube aspirate or bilious vomiting is diagnostic in the initial postoperative period. Kinking, hematoma, and edema are all signs of technical difficulty with the anastomosis. The late blockage is frequently associated with the internal volvulus of the distal jejunal limb [30].

Patients with symptoms need to get a CT scan. CT scans can indicate an internal hernia if the small bowel mesentery crosses the transverse colon mesentery and the jejunojejunostomy is located above the transverse colon. Additionally, the central mesenteric trunk to the right may exhibit crowding, stretching, and engorgement, as well as symptoms of SBO [33]. A swirling appearance of mesenteric fat or vasculature is the most accurate predictor of hernia, with a sensitivity of 80% and specificity of 90% [34].

Acute onset suggests complete blockage and frequently occurs after recent gastric bypass surgery with Roux-Y jejunojejunostomy; the patient has an acute post-surgical course characterized by hypotension for which no particular reason can be identified. At this point, the differential diagnosis includes occult bleeding, myocardial infarction, pulmonary collapse, pulmonary embolism, and infection. Serial hematocrit levels rule out bleeding; ECG and cardiac enzymes rule out myocardial infarction; and a chest X-ray rules out significant pulmonary collapse. The differential diagnosis, therefore, comes down to pulmonary embolus vs. an intra-abdominal disaster. Computed tomography (CT) can detect intra-abdominal pathologies at this stage, which is diagnostic [30]. CT imaging of the abdomen has limits when diagnosing internal hernias. According to Higa et al. [19], 20% of patients with internal hernias had negative CT scan results. If the CT scan is equivocal, patients continue to exhibit symptoms indicative of SBO. In that case, they should be investigated laparoscopically to rule out an internal hernia or another cause of SBO, and all three possible mesenteric defects should be examined.

Management strategies

Non-surgical Management

Gastric bypass surgery-associated bowel obstruction often requires emergency intervention, as delayed treatment can lead to poorer outcomes. Surgical intervention has become the primary approach in such cases; however, conservative treatment is also opted for in some cases, with positive outcomes. Several studies have shown the effectiveness of conservative treatment, including bowel rest, nasogastric decompression, and IV fluid hydration, in some cases. For example, Loftus et al., in their study, reported that four of 28 patients who received only conservative therapy did not require surgery. However, one patient was later readmitted and underwent surgery [35]. Other authors have also suggested opting for a conservative approach in cases of uncomplicated bowel obstruction [36,37]. Bowel rest primarily focuses on keeping patients at nil per os, which means that they are not allowed to consume any food or fluids to prevent further distension and to allow the bowel to rest. Lewis et al., in their study in which 13 patients had early jejunojejunostomy obstruction after laparoscopic gastric bypass, reported that 10 of the 13 patients were treated conservatively. The patients were treated with rehydration, rest, and continuous monitoring. No patient required intensive care unit (ICU) admission [38].

Similarly, another study concluded that surgical options are only sometimes necessary. In the study, almost half of the patients with jejunojejunostomy obstructions were treated with nonoperative treatment, which included bowel rest and nasogastric decompression [39]. However, operative intervention is always necessary in cases of narrowing the lumen. Similarly, RYGB-associated bowel obstruction generally requires surgical intervention. Even when bowel obstruction is relieved with conservative management, the causative factors, such as stenoses and adhesions, remain, which can lead to recurrent bowel obstruction.

Furthermore, ischemia usually takes around six hours to start and is followed by necrosis. Consequently, it can lead to gastric remnant blowout, which can have adverse consequences for patients. Ischemia is associated with a 30% mortality rate, which is much higher compared to 3% mortality in SBO without ischemia [40]. Furthermore, delays in surgical intervention can lead to adverse outcomes. Patients who present to the operating room within 24 hours of SBO are less likely to have bowel resection than those who presented after 24 hours (12% vs. 29%) [41].

The success of non-surgical management in the bariatric population is generally low, as the mechanical nature of most obstructions post bypass surgery usually necessitates surgical correction. Loar et al. presented a case study of a pregnant woman with a history of laparoscopic RYGB who presented with abdominal pain and a suspected SBO [42]. The CT scan showed bowel distention. She received conservative treatment with bowel rest and nasogastric decompression, which resulted in the resolution of symptoms. However, several days later, she presented with dark emesis and melanotic diarrhea. Sigmoidoscopy revealed no signs of obstruction. Her condition improved, but she had to undergo an emergent cesarean a few days later. During the procedure, the surgeons saw healthy small bowel loops. After three days postoperatively, she developed septic shock, accompanied by multi-organ failure. Exploratory laparotomy showed necrotic small bowel and right colon. The patient died after life support was removed. The autopsy showed ischemic necrosis along with bowel volvulus and perforation [42]. In cases of SBO post laparoscopic RYGB, the surgical option is the only viable option, and nasogastric decompression is usually ineffective [43].

Surgical Management

Surgical intervention is often the definitive treatment for bowel obstruction following gastric bypass surgery. The approach to surgery, whether laparoscopic or open, depends on the underlying cause of the obstruction. However, in most cases, the laparoscopic approach is practical. For example, Koppman et al. reported that 10 out of 11 patients with SBO after laparoscopic RYGB were treated with a laparoscopic procedure [44]. One patient who required open surgery had a mesenteric volvulus around a single adhesive band. Furthermore, there were also signs of ischemia that prompted open procedure [44]. Similarly, another study treated all the SBO cases laparoscopically [43]. Generally, the laparoscopic approach can be adopted in all patients unless the patient is hemodynamically unstable. If required, the procedure can be converted to an open procedure. For example, a retrospective study that included nine patients with bowel obstruction after a gastric bypass procedure reported that two patients underwent an open procedure. In one patient, the laparoscopic intervention was initially performed; however, the patient had an ischemic perforation in the Roux limb that prompted conversion to an open procedure [45].

During surgical intervention, especially in cases of SBO after RYGB, abdominal and pelvic regions should be examined thoroughly for any additional issues. A complete inspection of the small intestine is mandatory. The focus of the surgery should be to release any adhesions, close mesenteric defects, and clear obstructions [46]. The success of laparoscopic surgery largely depends on the surgeon's experience and the nature of the obstruction. However, laparoscopic procedures should be converted to open procedures if the obstruction is complex such as the presence of bowel ischemia or necrosis, or a history of multiple abdominal surgeries leading to dense adhesions [45]. The rate of conversion to open procedure is exceptionally high in bowel obstruction after laparoscopic RYGB. Champion and Williams, in their study, reported that two of 13 patients required conversion to open procedure. The main reasons were adhesion with ischemia in one case and internal hernia in another case [12].

Similarly, Nguyen et al. reported a conversion in two of the eight cases [22]. Husain et al. [14] also reported that conversion to open procedure was required in 19 of the 111 cases. Furthermore, bowel resection was needed in two cases. Apart from the underlying etiology, the decision for an open procedure is influenced by the patient's clinical condition at presentation and imaging findings.

Prognosis and long-term outcomes of bowel obstruction after gastric bypass surgery

One of the complications that could arise as a result of gastric bypass surgery is bowel obstruction; the outcomes rely on the location and timing of the obstruction.

While early postoperative obstructions often result from technical issues related to surgery, late obstructions may manifest months or even years later, typically associated with adhesions, internal hernias, or bowel strictures. The long-term prognosis of these patients majorly depends on the readiness for diagnosis and intervention. Late bowel obstruction occurs in 1-6% of cases, and 60% of these are due to internal hernias. Diagnosing an internal hernia is often difficult since the symptoms can be intermittent in presentation and may not show up on CT scans. Patients experience recurrent episodes of unexplained abdominal pain. While a CT sometimes helps by showing dilated small bowel loops or mesenteric vascular engorgement, it is not always definitive, especially for internal hernias in Petersen's space. In persistent cases, most of the time, exploratory laparoscopy is needed for diagnosis. To prevent this, it is crucial to close all mesenteric defects during surgery [47].

Prosthetic devices like silastic rings are commonly used in bariatric surgery. Smaller rings (4.5-5 cm) were found to cause erosion, and eventually, larger rings (7.5-8 cm) are now preferred; however, complications like small bowel herniation and obstruction have been reported. Early detection and removal of the silastic ring can prevent patient morbidity from small bowel ischemia [48].

The prognosis for gastric bypass depends on surgeons' systematic approach of thoroughly inspecting all potential herniation sites and avoiding missing other defects [49].

A single-center retrospective study analyzed 2,645 patients who underwent fully stapled laparoscopic Roux-en-Y gastric bypass (FS-LRYGB) from May 2004 to August 2008. Time taken during the procedure, hospital duration, readmission, re-operation, and early morbidity/mortality rates occurring within 30 days were then calculated. It revealed that the systematic approach and the complete standardization of the FS-LRYGB procedure contribute highly to their institution's low mortality and morbidity rates. This surgical procedure creates a long, narrow gastric pouch (5-6 cm) to reduce traction on the gastro-jejunostomy and minimize the risk of complications. Research suggests that long, narrow pouches may delay food transit and resist enlargement better than wider pouches. Hemorrhage was the most common in-hospital complication (3.42%); SBO occurred in 0.35% of patients, primarily due to hernias at the trocar site, a problem now resolved by using standard closure techniques [50].

Prevention of bowel obstruction post gastric bypass

Surgical Techniques

It has been well-studied that laparoscopic Roux-en-Y is safer and more effective. It offers clear benefits over open bariatric surgery, such as shorter hospital stays, fewer postoperative adhesions, and abdominal wall complications [22,51,52].

As per a randomized controlled trial by Puzziferri et al., there was a reduction in the incisional hernia rate in those who underwent laparoscopic gastric bypass [53]. However, Capella et al. [54] compared the incidence of bowel obstruction post laparoscopic vs. open approach gastric bypass surgery and found an unexpectedly higher incidence of bowel obstruction in the laparoscopic gastric bypass surgery group of patients due to the formation of intra-abdominal adhesions, which prevents loops of the bowel to slip into the mesenteric defects. While minimally invasive surgery reduces postoperative adhesions, it can also lead to a higher likelihood of internal hernias, which can cause SBO [19]. Hwang et al. [23] concluded in their study that the placement of the Roux limb (retrocolic vs. antecolic) seemed to affect the incidence of SBO. In particular, an antecolic positioning of the jejunal limb significantly decreased the risk of SBO. Multiple other studies have also shown that intestinal obstruction is more likely associated with retrocolic technique compared to antecolic technique [12,14]. Wu et al. [55], in their meta-analysis of 15 studies, established that closure of the mesenteric defect is more advantageous than non-closure during laparoscopic RYGB surgery and reduces the risk of SBO due to internal herniation.

Postoperative Monitoring

Permanent anatomical changes are made during RYGB surgery. Therefore, surgeons must understand the new post-procedural anatomy and the resulting physiological effects to make an effective and timely diagnosis of SBO postoperatively if a patient presents with clinical signs and symptoms. Husain et al. [14] stated that radiographic findings are not always reliable in making the diagnosis of SBO. However, using the upper gastrointestinal study series showed high sensitivity in diagnosing obstruction.

Regardless of the imaging modality used and its impression, if a patient presents with SBO symptoms like generalized abdominal pain, bloating, nausea, and vomiting post gastric bypass, they should be taken to the operating room for surgical exploration without any delay [15]. Lim et al. [56] noted that minimally invasive surgery could repair 70% of internal hernias causing obstruction. However, if laparoscopic repair becomes unsafe, surgeons should switch to open surgery. To avoid the risks of morbidity and mortality, it is essential to address the intestinal obstruction after gastric bypass surgery promptly and vigorously.

## Conclusions

Gastric bypass surgery, particularly RYGB, presents with significant postoperative complications at times that include internal hernias, adhesions, and stenosis. Prompt diagnosis and early surgical intervention are essential since delay can lead to necrosis. Surgical intervention remains the definitive treatment option that could include minimally invasive laparoscopic techniques that can be converted into open surgery based on complex cases. Preventive strategies could include antecolic rather than retrocolic Roux limb placement during the initial surgery. Future research should focus on optimizing surgical techniques and improving diagnostic modalities to minimize postoperative complications and enhance patient outcomes.
